# Hemp‐Derived Iron Oxide Nanoparticles for Biomedical Applications: Synthesis, Characterization, and Therapeutic Potential

**DOI:** 10.1002/open.202500189

**Published:** 2025-06-17

**Authors:** Nesrin Korkmaz, Rizvan İmamoğlu, Ahmet Karadağ, Ebru Şahin Yıldırım, Yusuf Ceylan, Fatih Şen

**Affiliations:** ^1^ Department of Basic Sciences and Health Hemp Institute Yozgat Bozok University Yozgat 66100 Türkiye; ^2^ Department of Molecular Biology and Genetics Faculty of Sciences Bartın University Bartın 74100 Türkiye; ^3^ Health Institutes of Türkiye (TUSEB) Türkiye Cancer Institute İstanbul 34718 Türkiye; ^4^ Department of Chemistry, Science and Letters Faculty Bursa Uludağ University Bursa 16059 Türkiye; ^5^ İmranlı Vocational School Department of Property Protection and Security Sivas Cumhuriyet University Sivas 58140 Türkiye; ^6^ Health Services Vocational School Sivas Cumhuriyet University Sivas 58140 Türkiye; ^7^ Sen Research Group Biochemistry Department Faculty of Arts and Science Dumlupınar University Evliya Çelebi Campus Kütahya 43100 Türkiye

**Keywords:** biological activity, *cannabis sativa* L. ( *Hemp*), green synthesis, hematite nanoparticles, iron oxide nanoparticles (IONPs), magnetite nanoparticles

## Abstract

Iron oxide nanoparticles (IONPs) have emerged as the most widely synthesized metal nanoparticles in sustainable chemistry due to their unique magnetic properties, excellent biocompatibility, biodegradability, and non‐toxicity. In this study, IONPs are successfully synthesized via a rapid, sustainable, and environmentally friendly green synthesis approach using *Cannabis sativa* L. leaf extract. X‐ray diffraction analysis determined that the synthesized NPs had an average particle size of 18.8 nm, while transmission electron microscopy images reveal a spherical morphology with sizes ranging from 12 to 21 nm. Fourier‐transform infrared spectroscopy analysis confirmed the presence of cannabinoids, terpenoids, and flavonoids, which are believed to play a crucial role in the formation and stabilization of IONPs. Its photocatalytic potential is demonstrated through the degradation of bromophenol blue dye. Additionally, the NPs exhibited significant antibacterial and antifungal activity against various microbial species, along with promising anticancer effects on cancer cell lines. In conclusion, this study provides a promising foundation for advancing the large‐scale, commercial production of IONPs through green synthesis methods. By offering an eco‐friendly and efficient alternative to conventional nanoparticle synthesis, the findings contribute significantly to the growing body of research in sustainable nanotechnology.

## Graphical abstract

Hemp‐derived iron oxide nanoparticles are synthesized via a green approach and characterized using various analytical techniques. These nanoparticles exhibit desirable physicochemical properties, including biocompatibility and magnetic responsiveness. The study explores their therapeutic potential in biomedical applications, highlighting their role in drug delivery, imaging, and anticancer activity.

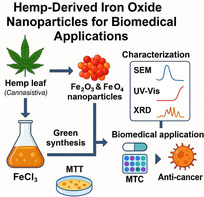

## 1 Introduction

Sustainability is a crucial global concern that directly affects environmental, economic, and societal well‐being. The integration of green chemistry principles into nanotechnology plays a pivotal role in reducing environmental hazards, lowering costs, and enhancing process safety. Metal oxide nanoparticles (MONPs), particularly iron oxide nanoparticles (IONPs), have gained significant attention due to their wide applications in environmental remediation and biomedical fields. However, conventional methods for synthesizing these nanoparticles often rely on toxic chemicals, energy‐intensive processes, and non‐renewable resources, which contradict the principles of green chemistry.^[^ ^1^, ^2^, ^3^ ^]^

Nanotechnology is the whole of science, engineering, and related technological studies by controlling matter at the atomic and molecular levels. Nano and nanotechnology are the technologies of the 21st century, and they are concepts that we come across in every aspect of our lives, and they now have a wide range of applications. It brings innovations in many fields such as medical sciences, materials, medicine, defense, textile, economy, computer technology, clean energy sources, sustainable energy, environment, and food.^[^ ^4^, ^5^, ^6^, ^7^, ^8^ ^]^ Using materials and materials effectively is one of the goals of nanotechnology and constitutes a holistic approach in terms of nanotechnology material. Nanotechnology enhances the durability of materials with its advanced properties, thereby minimizing the need for maintenance and repairs. Nanotechnology simplifies production by reducing the required steps. It provides a significant reduction in the protection of resources, raw materials, energy consumption, and, as a result, carbon dioxide emissions.^[^ ^7^, ^8^, ^9^, ^10^ ^]^ Many methods are used to synthesize metal nanomaterials (hydrothermal),^[^ ^11^ ^]^ sol‐gel,^[^ ^12^ ^]^ chemical reduction.^[^ ^10^, ^13^ ^]^ The use of biological resources, especially plant‐based materials, in nanoparticle synthesis offers an eco‐friendly and sustainable alternative. Hemp (*Cannabis sativa* L.) is an economically valuable and environmentally beneficial plant, widely utilized in various industries. However, the leaf part of the plant, which is considered agricultural waste, remains underutilized in high‐value applications such as nanomaterials production. This presents an important research gap in the current literature.^[^ ^14^, ^15^, ^16^, ^17^, ^18^, ^19^, ^20^, ^21^, ^22^, ^23^, ^24^, ^25^ ^]^ While numerous studies have investigated the green synthesis of metal nanoparticles using plant extracts, the use of hemp leaf waste biomass as a reducing and stabilizing agent in the synthesis of IONPs is still largely unexplored. Current research predominantly examines the flowers, seeds, and fibers of the hemp plant for industrial and medical applications, overlooking the potential of the leaves, which are often treated as waste. Additionally, very few studies comprehensively investigate the multifunctionality of such nanoparticles in a single study—covering structural, photocatalytic, antimicrobial, and anticancer properties.

**Figure 1:**
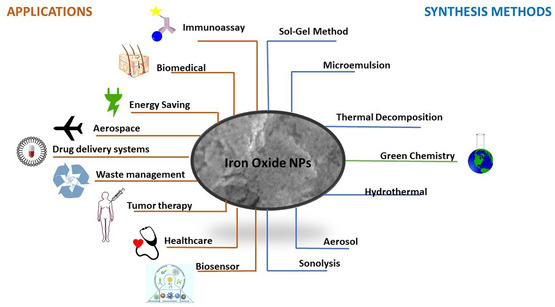
An overview of the technological application areas and synthesis pathways of IONPs.

Iron oxide (IO) compounds are widely found in nature and can be easily synthesized in the laboratory. There are sixteen different types of IO. The most common and most important IOs in nature: goethite, akaganeite, lepidocrocite, magnetite, and hematite.^[^ ^26^, ^27^ ^]^ The size of ferromagnetic IONPs is smaller than 20 nm and exhibits extraordinary superparamagnetism. Owing to these properties, such oxides play a crucial role in technology (**Figure** **1**). The superparamagnetic nature of IONPs, along with their high surface area and minimal toxicity, has led to their use in biomedical applications, particularly in thermal therapy, MRI, and drug delivery.^[^ ^28^, ^29^, ^30^, ^31^ ^]^ In addition to these, Fe_3_O_4_ and Fe_2_O_3_ are among the agents to fight against environmental pollution, such as treating textile waste, especially dye.

This study aims to fill the identified gap by addressing the following objectives: 1) To develop an eco‐friendly, cost‐effective, and sustainable green synthesis method for iron oxide nanoparticles using Cannabis sativa L. leaf extract, a plant waste biomass. 2) To characterize the synthesized IONPs in terms of morphology, crystallinity, elemental composition, and particle size using techniques such as X‐ray diffraction (XRD), UV‐vis, Fourier‐transform infrared spectroscopy (FTIR), transmission electron microscopy (SEM), and esnergy‐dispersive X‐ray (EDX). 3) To evaluate the photocatalytic efficiency of the synthesized nanoparticles by examining their capacity to degrade bromophenol blue dye. 4) To investigate the biological properties of the IONPs, including: Antibacterial and antifungal activity, Anti‐biofilm potential, Anticancer effects on HeLa and HT‐29 cell lines. 5) To highlight the valorization of agricultural waste in nanotechnology applications and promote bio‐based innovation as a part of environmental sustainability strategies.

This research provides a holistic and innovative approach to nanoparticle synthesis that contributes to: Waste valorization by utilizing hemp leaves that would otherwise be discarded, Environmental sustainability by replacing toxic synthesis methods with green chemistry approaches, Nanotechnology development by producing multifunctional nanoparticles with potential in environmental cleanup and biomedicine, Sustainable industry practices through scalable, low‐cost synthesis methods using abundant biomass.

The outcomes of this study are expected to inspire further research into agro‐waste utilization, bio‐based nanomaterials, and circular economy models in the field of sustainable nanotechnology.

## 2 Experimental Section

### 2.1 Preparation of the Plant Extracts

The plant extract necessary for the synthesis reaction was obtained from hemp leaves. The ripe leaves were collected from the grounding area, washed with pure water and cleaned of dust and foreign substances. The leaves have been completely dried without exposure to direct sunlight and in an environment with good air circulation. 60 g of dried hemp leaves were weighed and heated at 80 °C for 1 h by adding 1 L of distilled water to it. After the process, the mixture was cooled to room temperature and the extract obtained by filtering was stored at +4 °C for use in nanoparticle synthesis.

### 2.2 Green Synthesis of IONPs

**Scheme 1:**
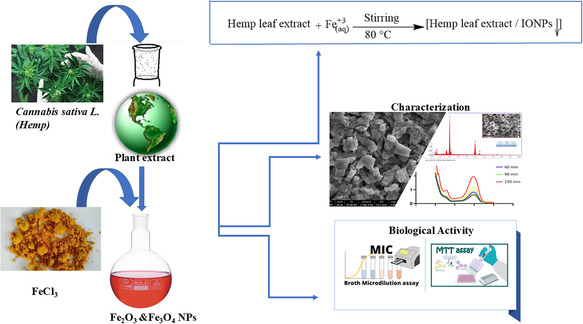
A schematic plan of the “green synthesis” of IONPs and subsequent work.

All chemicals are of analytical purity and were obtained from Sigma‐Aldrich (FeCl_2_·4H_2_O ≥ 99, FeCl_3_·6H_2_O ≥ 97). Iron nanoparticle synthesis was performed by using the synthesis methods in literature.^[^ ^32^, ^33^ ^]^ Using hemp extract, Fe^3+^ and Fe^2+^ ions were added to pure aqueous hemp extract in a ratio of 2:1 mole and a yellowish heterogeneous solution was obtained. Then, 1.0 M NaOH solution was added to the solution drop by drop until the pH reached 11. The mixture was stirred continuously at 80 °C for 3 h. In this process, the color of the solution darkened, indicating the formation of iron nanoparticles. The synthesized nanoparticles were separated with the help of a magnet, washed several times with distilled water and centrifuged. The resulting product was dried at 90 °C for 12 h and prepared for characterization processes. **Scheme** **1** briefly summarizes this work.

### 2.3 Characterization of NPs

The average particle diameter and crystal structure of the synthesized iron nanoparticles were analyzed with the Panalytical EMPYREAN model multipurpose XRD meter (MP‐XRD) located at Yozgat Bozok University. The surface morphology and elemental composition of the sample were determined using the QUANTA FEG 450 model field emission environmental scanning electron microscope‐energy distribution spectrometer (FE‐ESEM‐EDX). In addition, FTIR was performed with Two Perkin Elmer Spectrum ATR devices in the wavelength range min 400–4000 cm^−1^ to examine the surface properties.

### 2.4 Photocatalytic Activity

Bromophenol blue (BPB) dye solution was prepared at a concentration of 1 mg mL^−1^ for the photocatalytic activity test. Prior to the addition of nanoparticles, a control measurement was performed by recording the absorbance of the dye solution without nanoparticles. Following this initial control, 0.2 g L^−1^ of nanoparticles was added to the dye solution and the mixture was continuously stirred under sunlight. Absorbance values were then recorded at defined time intervals (30, 60, 90, and 300 min) to monitor the photocatalytic degradation of the dye. This setup enabled a comparison between the control (dye‐only) and nanoparticle‐treated samples to evaluate the specific contribution of the nanoparticles to dye degradation.

### 2.5 Antifungal and Antimicrobial Studies

The antimicrobial and antifungal effects of nanoparticles were determined using the minimal inhibitory concentration (MIC) test. Gram‐positive (*Staphylococcus aureus* and *Enterococcus faecalis*), gram‐negative (*Klebsiella pneumonia* and *Escherichia coli*) bacterial and fungal strains (*Cladosporium utilis* and *Candida albicans*) were used in the test. Cultures taken from frozen stock cells were adjusted to 0.5 McFarland units and a serial dilution method was applied with nanoparticles at a concentration of 1 mg mL^−1^ on 96‐well plates. The cells were incubated with the nanoparticles at 37 °C for 24 h. The MIC results were evaluated by spectrophotometric absorbance measurements.

### 2.6 Determination of Anticancer Feature

To evaluate the anticancer effects, the synthesized nanoparticles were analyzed by MTT test in HeLa and HT‐29 cancer cell lines. The cells were planted in 96‐well plates at a density of 5 × 10^4^ cells/well and incubated overnight at 37 °C in an atmosphere containing 95% humidity, 5% CO_2_ cytotoxicity was assessed by standard MTT test after 24‐h exposure. Absorbance readings are measured at 570 nm by a microplate reader (Thermo Instruments, Inc.) were taken with. The effects of nanoparticles at different concentrations (100, 50, 25, 12.5 μL) were tested and IC_50_ values were calculated. Cytotoxicity percentage and IC_50_ calculations were evaluated according to control groups using GraphPad Prism 8.01 software.^[^ ^10^ ^]^

## 3 Results and Discussion

### 3.1 XRD Analysis of IONPs

**Figure 2:**
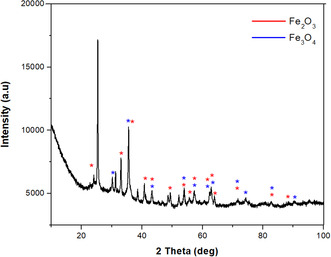
XRD pattern of Fe_3_O_4_&Fe_2_O_3_ NPs.

As a result of XRD analysis, it is seen that NPs synthesized by biogenic reduction are synthesized as a mixture of Fe_3_O_4_&Fe_2_O_3_ (**Figure** **2**). The XRD data, as shown in Figure 2, indicates that the crystal planes of (220), (311), (400), (422), (511), (440), and (533) corresponding to the 30.13°, 35.54°, 43.25°, 54.05°, 57.28°, 62.86°, and 74.25° indicates the formation of Fe_3_O_4_ NPs. The data obtained showed that the space group of Fe_3_O_4_ is Fd‐3 m, and the crystal system is cubic (ICDD card no. 98‐008‐5806).

Characteristic peaks of Fe_2_O_3_ nanoparticles can be recognized at 2*θ* positions = 23.98° (012), 33.07° (104), 35.67° (110), 40.87° (113), 49.19(024), 53.87° (116), 57.24(122), 62.19° (214), 64.01° (300), and 71.54° (101). It was observed that the peaks of Fe_2_O_3_ were consistent with the rhombohedral crystal system (JCPDS no. 98‐016‐1291). Dense and sharp peaks undoubtedly revealed that Fe_3_O_4_&Fe_2_O_3_ NPs formed by the phytochemicals in *C. sativa* leaf extract acting as reducing agents are crystalline. The characteristic peaks of Fe_3_O_4_&Fe_2_O_3_ NPs are in agreement with the peak values of other Fe_3_O_4_&Fe_2_O_3_ NPs synthesized by the green synthesis method in the literature^[^ ^34^ ^]^; Ruíz‐Baltazar et al. 2020).

**Figure 3:**
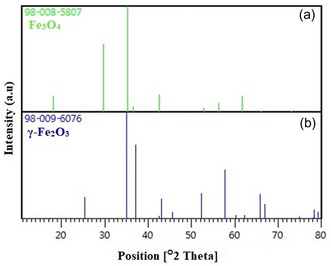
XRD peak list of Fe_3_O_4_ a), and γ‐Fe_2_O_3_ b).

Fe metal is an easily oxidized metal. There is usually a rapid conversion from zero value Fe to Fe_2_O_3_. Therefore, there is a possibility that the phases we obtain are not just hematite and magnetite, but a mixture of IOs. For this, comparisons were made with the standard XRD models of Fe_3_O_4_ and γ‐Fe_2_O_3_ (**Figure** **3**). The peaks obtained because of the analysis coincide with the standard magnetite XRD values with JCPDS file no: 98‐008‐5807 (Figure 3a), which reports the crystallographic values of the cubic crystal structure. Additionally, the obtained NPs were confirmed to be Fe_3_O_4_ and when compared with standard XRD values (Figure 3). As a result of this comparison, the values obtained as a result of XRD analysis showed that pure Fe_3_O_4_&Fe_2_O_3_.

The crystal size of the IONPs was calculated by substituting the obtained XRD data in the Debye–Scherrer formula (Equation (1)).

(1) $$d=\frac{K\lambda }{\beta \cos\theta }$$

In the equation, *d* is the crystal size, *K*(≈0.94): Scherrer's constant, *λ* (1.540 A°): the X‐ray wavelength, and *β:* the full‐width half‐maximum of the diffraction peak. The average particle size of the nanoparticles obtained due to the calculation was 18.8 nm. It has been reported in the literature that the particle size of NPs obtained using the chemical synthesis method was found to be 7.1 using the Debye–Scherrer formula.^[^ ^35^ ^]^

### 3.2 Scanning Transmission Electron Microscopy (SEM) Analysis of IONPs

**Figure 4:**
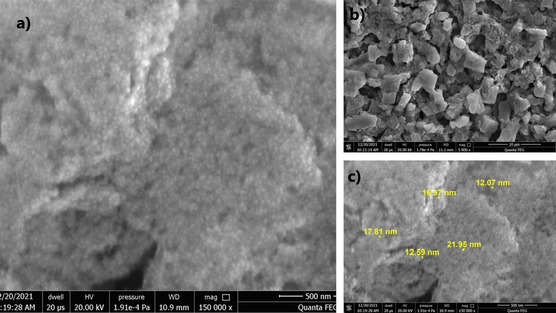
SEM image of IONPs by the biological method. a) Surface morphology of IONPs, b) Aggregated nanoparticle structures, c) Particle size measurement indicating a size range between 12.07 and 21.95 NM.

The shapes of IONPs produced using herbal agents are shown in **Figure** **4**. Due to the natural magnetism and Van der Waals forces of Fe_3_O_4_ NPs, they tend to form integrated clusters (Figure 4).^[^ ^36^ ^]^ Therefore, as can be seen from the SEM images, the NPs are integrally all‐in.

When the images were taken at 500 (Figure 4a,c) nm and 20 μm (Figure 4c) are examined, it is seen that small nanoparticles form clusters as a result of centrifugation and form larger cube like structures. According to the labels in the images, it is seen that the nanoparticles are in different sizes from each other. It has been reported in the literature that Fe_3_O_4_ NPs synthesized by hydrothermal and chemical synthesis methods have spherical‐shaped NPs below 10 nm.^[^ ^37^ ^]^

### 3.3 EDX Analysis of IONPs

**Figure 5:**
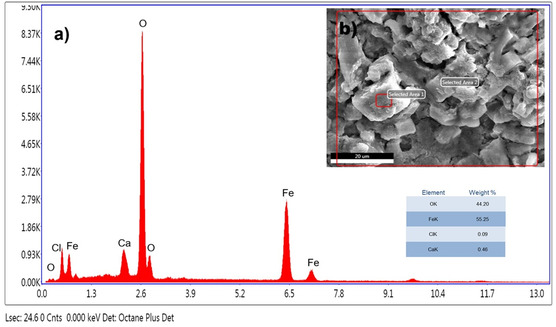
EDX analysis of IONPs prepared by the biological method.

EDX analysis was performed to evaluate the elemental composition of IONPs. The formation of IONPs (**Figure** **5**b) was demonstrated by the detection of a significant number of peaks for Fe and O (Figure 5a). The composition of the particles and the purity level of the particles in the biogenically synthesized IONPs were analyzed. We show that *C. sativa* mediated IONPs have 55.25% iron and 44.20% oxygen, respectively (Figure 5). Both iron and oxide peaks confirm the formation of IONPs. In addition, trace amounts of Cl and Ca originating from plant residues were reported in the EDX analysis.

The presence of chlorine in EDX spectroscopy was also commonly found as impurity in other studies synthesized by the green synthesis method. It has been observed that these impurities in the elements will be removed by washing the NPs with ethanol solvent.^[^ ^38^, ^39^ ^]^

### 3.4 UV‐Vis Spectral Analysis of IONPs

**Figure 6:**
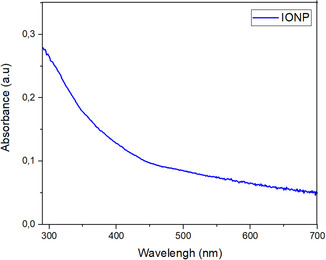
UV‐Vis spectrum of Fe_3_O_4_&Fe_2_O_3_ NPs.

UV‐Vis spectroscopy was performed on a diluted solution of colloidal IONPs and the resulting spectrum is presented in **Figure** **6**. No specific absorption peak was observed in the spectral analysis, but it was reported to exhibit a continuous absorption in the visible region between 280–700 nm. This absorption is attributed to the formation of IONPs. Similar spectral features have been reported in some studies in the literature.^[^ ^33^, ^40^, ^41^ ^]^

At the beginning of the experimental study, Fe^3+^ ions were reduced by the phytochemicals contained in the plant extract and turned into zero‐valent Fe^0^ particles. However, these particles are unstable due to the high reactivity of iron. Under experimental conditions, Fe^0^ particles were oxidized to form Fe^3+^/Fe^2+^ ions. In the study, the aggregation process following nucleation resulted in the formation of Fe_3_O_4_ and Fe_2_O_3_ nanoparticles, which were stabilized by the phytochemicals in the plant extract.

### 3.5 FTIR Analysis

The absorption bands observed in the plant extract at 3322.21, 2974.91, 2928.78, 1650.79, 1382.83, 1330.91, 1273.37, 1084.66, 1043.02, and 879.14 cm^−1^ correspond to various molecular vibrations. These include stretching of O—H and C—H bonds, C—O and C—C bonds in carbonyl groups, vibrations of saturated aldehyde, C—N stretching in amide groups, vibrations of C—C and C—H bonds in aromatic ring and bending of C—O bonds in phenolic compounds. These vibrations indicate the presence of organic compounds in the plant extract .^[^ ^42^ ^]^

**Figure 7:**
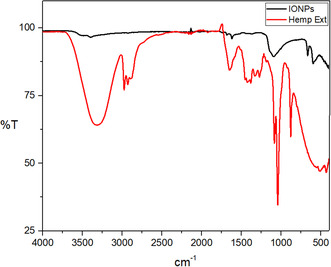
FTIR spectra of biologically derived IONPs and Hemp extract.

FTIR of IONPs‐*Hemp*. (**Figure** **7**) the peaks absorbed by IONPs and attributed to ‐OH appear as broad and strong bands at 3494–3397 cm^−1^. The peak at 665 and 599 cm^−1^ gives the Fe—O bond vibration of IONPs.^[^ ^35^, ^43^ ^]^ The peak observed at 1092 cm^−1^ gives the Fe—OH structural vibration. The peaks at 1684 and 1620 cm^−1^ are attributed to C—H vibrations. The peaks extending in the region between 400 and 600 cm^−1^ correspond to the characteristic peaks of IONPs.^[^ ^43^ ^]^

### 3.6 The Photocatalytic Effect of IONPs

**Figure 8:**
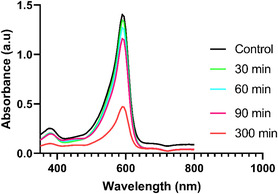
Photocatalytic activity of IONPs on the degradation of Brompenol Blue prepared by Biogenic method.

Increasing life expectancy, population growth, and the need for new industrial products have significantly increased the industrial growth rate. However, wastewater resulting from industrial activities containing toxic chemicals and organic dyes has become a problem that poses a serious threat to human health. In this study, the photocatalytic degradation properties of organic dye such as Bromophenol Blue were investigated to evaluate the effectiveness of biogenically synthesized IONPs (**Figure** **8**).

**Graph 1:**
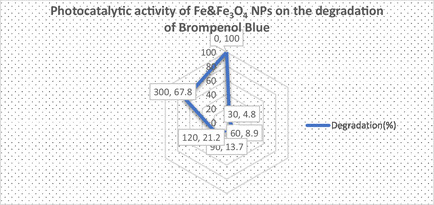
Photocatalytic activity of IONPs on the degradation of Brompenol Blue.

The percentage calculation was calculated according to the formula below and the results are shown in **Graph** **1**.

(2) $$\%\text{Degradation}=\frac{\text{Ao}‐A}{\text{Ao}}*100$$

The results of the study showed that the dye gradually degrades over periods of 0 to 150 min. A significant color change has been observed that occurs from a dark medium to a colorless solution. The photocatalytic effect of IONPs prepared by the green synthesis method was tested at time intervals of 0, 30, 60, 90, and 300 min, and it was determined that the optical density decreased gradually to 1.39, 1.33, 1.26, 1.15, and 0.47 values, respectively. Previous studies have revealed that plant phytochemicals act as reducing agents and increase photocatalytic activity by acting as catalysts in this process.^[^ ^44^, ^45^ ^]^

### 3.7 Biological Activity

#### 3.7.1 Minimum Inhibitory Concentration (MIC) Assay

The minimum inhibitory concentration (MIC) method was employed to assess the antibacterial and antifungal efficacy of green‐synthesized IONPs against various microorganisms. Tested strains included Gram‐positive bacteria (*Staphylococcus aureus, Enterococcus faecalis*), Gram‐negative bacteria (*Klebsiella pneumoniae, Escherichia coli*), and fungi (*Candida albicans, Candida utilis*). As indicated in **Table** **1**, the MIC values for bacterial strains ranged from 50 to 80 μg mL^−1^, demonstrating the nanoparticles’ potent antimicrobial activity. This effect is attributed to reactive oxygen species (ROS) generation induced by oxidative stress from the IONPs.^[^ ^46^, ^47^, ^48^ ^]^

**Table 1:** Antibacterial and antifungal MIC results.

Microorganisms	MIC Values of IONPs [μg mL^−1^]
*E. coli*	278 ± 22
*K. pneumoniae*	363 ± 21
*S. aureus*	399 ± 19
*E. faecalis*	277 ± 12
*C. albicans*	393 ± 22
*C. utilis*	275 ± 21

A review of the literature reveals extensive research on the antimicrobial properties of iron‐based nanoparticles. Studies confirm that green‐synthesized IONPs exhibit comparable efficacy against both Gram‐negative (e.g., *E. coli*) and Gram‐positive (e.g., *S. aureus*) bacteria.^[^ ^49^, ^50^ ^]^ Similarly,^[^ ^51^ ^]^ demonstrated that IONPs could serve as a promising antimicrobial agent, particularly against *E. coli*.

#### 3.7.2 Evaluation of Cell Survival and Death

**Figure 9:**
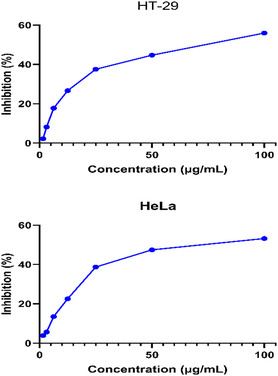
Cytotoxicity effect of green‐synthesized IONPs.

Cytotoxic effects have been investigated on the human colorectal adenocarcinoma cell line HT‐29 and the human cervical carcinoma cell line HeLa. The cytotoxicity effects of IONPs obtained by the green synthesis method are summarized in **Table** **2** and visualized in **Figure** **9**.

**Table 2:** Cytotoxicity effect of green‐synthesized IONPs NPs.

Cell Line	IC_50_ of IONPs [μg mL^−1^]
*HeLa*	65 ± 6
*HT‐29*	63 ± 5

Metal nanoparticles enter the cell by disrupting the structure of the plasma membrane and lead to oxidative stress, downregulation of anti‐apoptotic proteins and the formation of apoptosis in cancer cells.^[^ ^52^ ^]^ In this study, IC_50_ values for HeLa and HT‐29 cell lines were determined as 64.64 and 63.25 μg mL^−1^, respectively. There are various studies in the literature that iron nanoparticles have cytotoxic effects on cancer cells.^[^ ^53^, ^54^, ^55^ ^]^ Alangari et al. In a study conducted in 2022, it showed that it significantly inhibited the growth and spread of cancer cell lines.^[^ ^56^ ^]^

## 4 Conclusion

In this study, IONPs were successfully synthesized via a single‐step green approach using *C. sativa* leaf extract as the sole reducing and stabilizing agent, eliminating the need for secondary chemicals. The nanoparticles were comprehensively characterized through SEM, EDX, XRD, UV‐Vis, and FTIR spectroscopy. XRD analysis confirmed the high purity of the IONPs, while SEM imaging revealed a particle size distribution of 12–21 nm, with notable agglomeration and low crystallinity, consistent with an amorphous nanostructure (18.8 nm). EDX quantification indicated a ≈99% formation efficiency for the green‐synthesized IONPs.

The catalytic potential of the nanoparticles was demonstrated by the degradation of bromophenol blue dye, evidenced by a progressive reduction in absorbance at 590 nm. Beyond their antimicrobial efficacy against bacterial (e.g., *S. aureus, E. coli*) and fungal (e.g., *C. albicans*) pathogens, the IONPs exhibited dose‐dependent cytotoxicity against HeLa and HT29 cancer cell lines, suggesting therapeutic applicability. Furthermore, their utility in wastewater treatment and industrial dye degradation highlights their versatility in environmental remediation.

To fully harness these functionalities, further investigations are necessary to optimize dosage, reaction kinetics, and long‐term stability. Collectively, these findings underscore the potential of eco‐friendly IONPs for biomedical and environmental applications, aligning with sustainable nanotechnology paradigms.

## Conflict of Interest

The authors declare no conflict of interest.

## Author Contributions

**Nesrin Korkmaz**: formal analysis (equal); methodology (equal); validation (equal); writing—original draft (equal). **Rizvan Imamoglu**: methodology (equal); writing—original draft (equal); writing—review and editing (equal). **Ahmet Karadağ**: formal analysis (equal); writing—review and editing (equal). **Ebru Şahin Yıldırım**: Contributed to nanoparticle synthesis and XRD, UV‐vis analyses, **Yusuf Ceylan**: Contributed to antimicrobial activity testing and interpretation. **Fatih Sen**: formal analysis (equal); writing—review and editing (equal). funding acquisition (equal); project administration (equal); supervision (equal).

## Data Availability

The data that support the findings of this study are available from the corresponding author upon reasonable request.
